# Ex‐ante evaluation of promising soybean innovations for sub‐Saharan Africa

**DOI:** 10.1002/fes3.172

**Published:** 2019-05-16

**Authors:** Sika Gbegbelegbe, Arega Alene, Alpha Kamara, Keith Wiebe, Victor Manyong, Tahirou Abdoulaye, Petros Mkandawire

**Affiliations:** ^1^ International Institute of Tropical Agriculture (IITA) Lilongwe Malawi; ^2^ International Institute of Tropical Agriculture (IITA) Kano Nigeria; ^3^ International Food Policy Research Institute Washington District of Columbia; ^4^ International Institute of Tropical Agriculture (IITA) Dar es Salaam Tanzania; ^5^ International Institute of Tropical Agriculture (IITA) Ibadan Nigeria

**Keywords:** climate change, geospatial bio‐economic modeling, IMPACT, import dependency, soybean, sub‐Saharan Africa

## Abstract

This study undertakes an ex‐ante evaluation of the effects of alternative technology and policy options on soybean supply and demand in sub‐Saharan Africa (SSA) to 2050. Current soybean consumption in SSA is dominated by cooking oil followed by soybean cake used as animal feed. Due to weak processing sectors and low soybean yields, the region is currently importing about 70% of its consumption requirements. Based on the results from a geospatial bio‐economic modeling framework, soybean consumption in SSA is projected to more than double by 2050 compared to 2010 due in part to a rising population and rising incomes. On the other hand, supply from domestic production is projected to increase by 80% over the same period. Hence, by 2050, net imports into SSA would be nearly 4 times higher than supply from domestic production. Under a future drier climate, some of the production gains achieved through soybean research and extension would be lost and this would further worsen the soybean demand gap in SSA relative to the baseline. This study shows that relying on conventional breeding alone to increase soybean yields in SSA would not be enough to substantially reduce the future demand gap. A combination of promising innovations affecting the soybean value chain across SSA would be needed to close the soybean demand gap in SSA by 2050 under a drier future climate.

## Graphical abstract

Sub‐Saharan Africa currently spends more than US$ 1 billion on net imports of soybean‐based agricultural products. The soybean demand gap for this region is projected to increase in the future. This study shows that a combination of promising innovations affecting the soybean value chain across SSA would be required to close the future soybean demand gap.

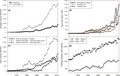

## 1 INTRODUCTION

Sub‐Saharan Africa (SSA) spends more than US$ 1 billion on net imports of soybean‐based agricultural products; this is more than the region's public agricultural expenditures which stood at about US$ 812 million in 2011 (ASTI, 2017). In addition, current expenses on soybean imports are very close to the amounts spent during the global food price spike of 2007/8 when the value of net imports of soybean‐based products in SSA increased from US$ 0.6 billion in 2006 to US$ 0.9 billion in 2007 and US$ 1.4 billion in 2008.

Some countries in SSA are currently trying to strengthen their domestic soybean industry in an attempt to enhance food safety and also bring about income growth opportunities where they have a comparative advantage. In 2004, Mozambique imported about 70% of its consumption requirements for broilers but a large portion of the imported broilers were past or near expiry date (Karnani & McKague, 2014). Since then, the country has transformed its poultry value chain and is now experiencing an increase in soybean production used as animal feed in the poultry sector (Karnani & McKague, 2014). In recent years, South Africa has been strengthening its soybean cake processing sector and in return has seen dramatic increases in its soybean acreage and production (Esterhuizen, 2016). Similarly, Nigeria has been implementing various measures over the years to enhance its domestic production of soybean cooking oil for human consumption and soybean cake targeted mainly for the poultry industry (Nzeka, 2014).

This paper discusses the current status of soybean in SSA and explores options which could be used to reduce import dependency for soybean in SSA under climate change; it is the first paper to analyze plausible futures for soybean production, consumption, and trade in SSA under climate change. The very few studies which have analyzed plausible futures for oilseeds and for soybean in particular in SSA have usually assumed soybean to be insignificant among oilseeds in SSA (Hartman, West, & Herman, 2011; Hirsch, 2002; Omamo et al., 2006).

## 2 BACKGROUND: CURRENT STATUS OF SOYBEAN IN SSA

Soybean consumption is spread more widely across countries in SSA unlike production which is dominated by South Africa and Nigeria. The latter accounts for about 29% of total production, whereas South Africa accounts for 40% (Table 1).

**Table 1:** The top soybean producers and consumers in Africa—average between 2009 and 2011

Country	Grain production (000 MT)	Total consumption (000 MT)	Grain consumption from net imports of oil and meal* (000 MT)	
			Grain from net oil imports	Grain from net meal imports
South Africa	597	2,872	1,357	1,216
Nigeria	425	453	14	27
Zambia	116	137	52	0
Malawi	78	161	86	2
Zimbabwe	76	257	52	0
Rwanda	50	55	4	0
Angola		463	455	1
Senegal		376	363	7
Mozambique		217	149	67
Mauritius		164	114	50
Madagascar		128	125	2
Mauritania		118	118	0
Tanzania		81	76	0
SSA	1,480	5,941		

* Additional grain from oil and meal for animal feed is computed as: (total consumption of oil/meal minus domestic production)/conversion coefficient; with the conversion coefficient to grain equivalent being 0.18 for cooking oil and 0.73 for soybean meal; where “total consumption of oil/meal minus domestic production” is lower than zero, additional demand is zero; note “total consumption of oil/meal minus domestic production” = “net imports − stocks”.

*Source:* Authors' computations using data from FAOSTAT (2017); since latest FAOSTAT data are usually updated, we use FAOSTAT data till 2011.

**Figure 1:**
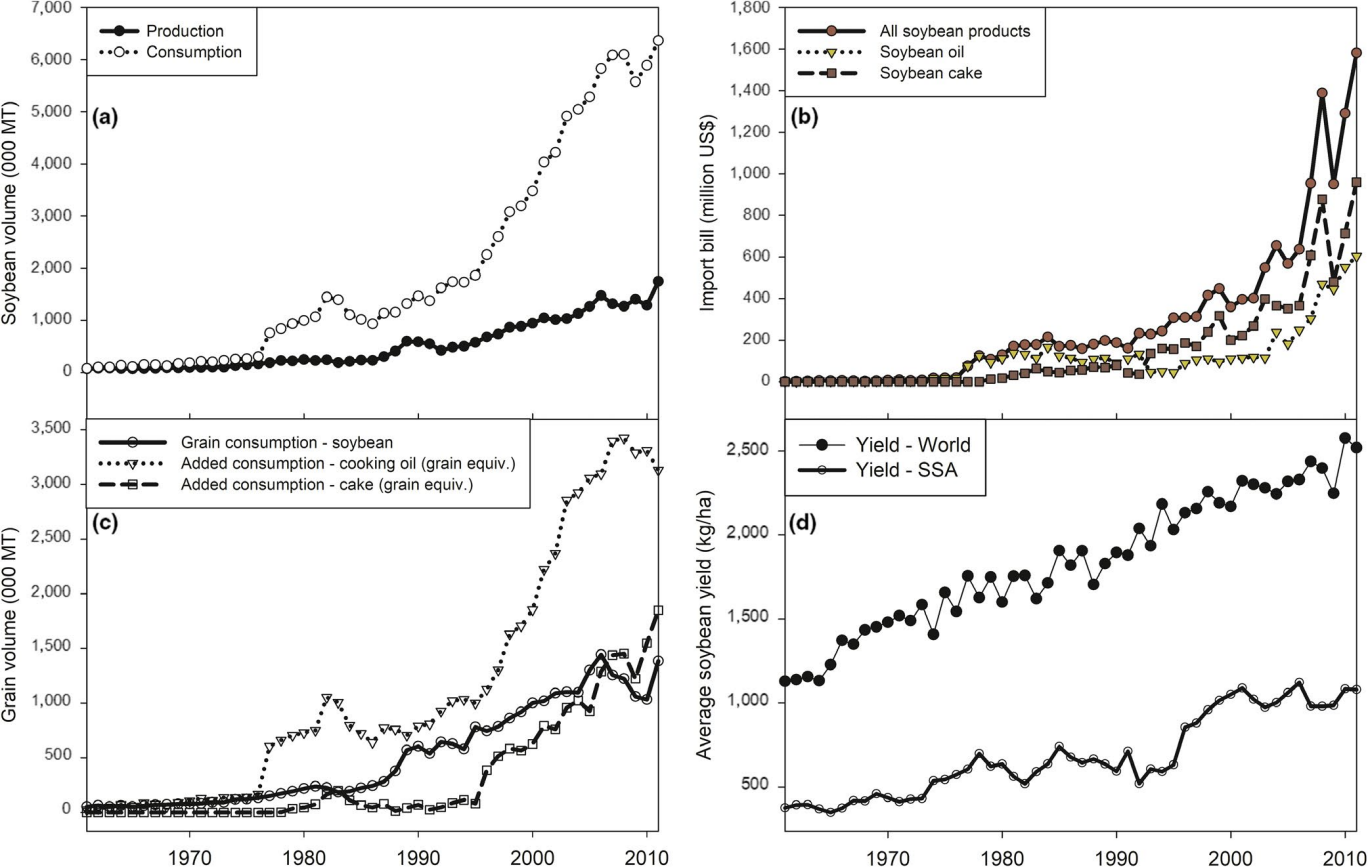
Past trends in (a) soybean production and consumption in SSA; (b) soybean import bill in SSA; (c) soybean consumption patterns in SSA; and (d) soybean yields in SSA. *Source*: Authors' computations using data from FAOSTAT (2017)

In 2011, SSA produced 1.7 million t of soybean grains but consumed 6.3 million t (Figure 1a). Hence, the demand gap, which can be filled only through imports and stocks, stood at 72% of total consumption. Soybean consumption in the region is currently dominated by cooking oil: the net imports of cooking oil alone, when converted in grain equivalents, account for 55% of total soybean grain consumption in the region (Figure 1c). This additional soybean consumption (net imports of cooking oil) has surged in SSA since 1995 and has been the main driver in total consumption (Figure 1a,c). Similarly, the additional demand for soybean cake (net imports of cake) used for animal feed has risen substantially over the years, fueled by the growing poultry industry to meet the increasing demand for meat in SSA. The rising demand for soybean cooking oil and cake implies that domestic grain producers and processors would need to more than double their outputs to meet current consumption requirements in SSA.

Apart from challenges faced by the soybean processing sector which is not producing enough to meet the rising demand, soybean grain production remains low across SSA. The crop is affected by various stresses, both abiotic such as weather extremes and low soil fertility, and biotic such as diseases, insect pests, and weeds (Hartman et al., 2011). Additional constraints include poor nodulation, pod shattering, poor seed viability, poor agronomic practices, and inadequate access to improved seeds for farmers. These constraints partially explain why soybean yields in SSA are less than 50% that of the world average (Figure 1d).

### 2.1 Initiatives to tackle constraints to soybean production in SSA

International soybean breeding involving the International Institute of Tropical Agriculture (IITA) and its partners has been instrumental in tackling some of the constraints to production in Africa. By 1995, IITA and its partners had developed improved promiscuous soybean lines with good seed viability, varying maturity lengths, and one or two additional traits including resistance to pod shattering and frogeye leaf disease (Tefera, 2011). In the mid‐2000s, the Institute and its partners were able to develop improved soybean germplasm with a combination of traits which increased yields by 25%–30% on field trials conducted in research stations. The improved lines were highly promiscuous with good seed viability, varying maturity lengths to escape drought, and the following additional traits: resistance to pod shattering and high resistance to rust, bacterial pustule, and frogeye leaf spot disease.

As of now, IITA and its partners in the National Agricultural Research and Extension Systems (NARES) are using two approaches to enhance soybean yields on farmers' fields in Africa: international breeding to develop improved germplasm and sustainable production systems with a focus on developing innovative agronomic practices for yield increase. Some of the improved agronomic practices include the use of inoculants and increasing soybean plant population (Kamara, Ewansiha, Boahen, & Tofa, 2014).

## 3 METHODOLOGY AND DATA

**Figure 2:**
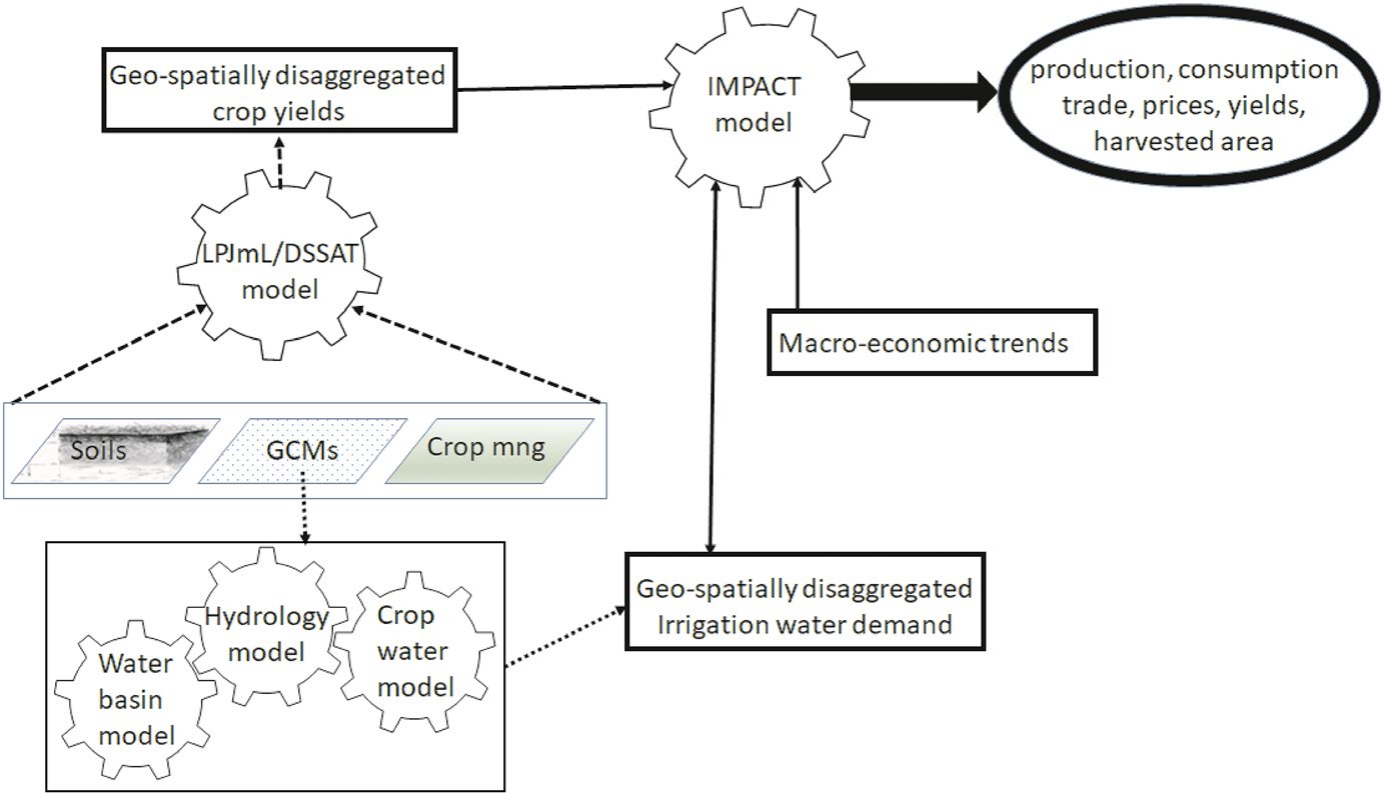
Spatial bio‐economic modeling framework. *Source*: Adapted from Islam et al. (2016) and Robinson et al. (2015)

The methodological framework used in this study involves a combination of geospatial crop modeling, hydrology modeling, and economic modeling (Figure 2). Geospatial crop modeling is used to quantify the impact of global climate models on crops' biophysical yields; it involves the use of the Decision Support System for Agrotechnology Transfer (DSSAT) or the Lund–Potsdam–Jena managed Land (LPJmL) models (Bondeau & Smith, 2007; Jones et al., 2003). For this study, we use the LPJmL model, a global gridded crop model which simulates biogeochemical and biophysical processes linked to land use for crops, pasture, and natural vegetation (Bondeau & Smith, 2007). For soybean and other crops, the model uses crop functional types which are simulated plant prototypes adapted to various climates and which capture key traits including. The LPJmL model has been successfully validated across multiple sites relative to crop yields, sowing dates, and a range of other biophysical and biogeochemical indicators. For soybean specifically, the LPJmL has been validated for the crop fraction of photosynthetically active radiation on a global scale and seasonal CO_2_ flux in Bondville (Illinois, USA) (Bondeau & Smith, 2007). The model was recently used in conjunction with the DSSAT model to quantify the impact of climate change on global crop productivity (Müller & Robertson, 2014). For soybean, both the LPJmL and DSSAT models projected a reduction in soybean yields across SSA due to climate change; however, the yield reductions were slightly more pronounced under the LPJmL model (Müller & Robertson, 2014).

For the purpose of linking the LPJml model with economic models, the production of soybean and other crops was simulated for two periods, a base period around 2000 and a future period around 2050; annual yield growth rates between “2000” and “2050” were also provided (Müller & Robertson, 2014). For the base period, crop productivity was simulated yearly between 2000 and 2009 using daily weather data from the Inter‐Sectoral Impact Model Intercomparison Project (ISI‐MIP); the yields were then averaged across years to obtain the approximate yields for the base year, “2000.” For future crop productivity under climate change, daily weather data from future climate models were taken from the ISI‐MIP and used to simulate yearly crop productivity from 2010 to 2059. Crop yields were then averaged between 2050 and 2059 to obtain approximate yields for 2050. All simulations were conducted at the 0.5‐degree resolution across the globe. Gridded soil data were taken from the Harmonized World Soil Database, whereas crop management patterns were captured through national cropping intensity calibrated using statistics from FAO (Müller & Robertson, 2014).

The hydrology model in IMPACT is used to estimate irrigated water demand under different general circulation models (GCMs) which reflect future climate models. Outputs from the crop and hydrology models are inputted into the International Model for the Policy Analysis of Agricultural Commodities and Trade (IMPACT), an economic model which uses various functional relationships and assumptions to project national and global food production, consumption, trade, and food security under alternative scenarios on population growth, income growth, and future climates (Robinson et al., 2015; Rosegrant & IMPACT development team, 2012; Rosegrant et al., 2008).

The IMPACT model version 3.2 was adjusted for the purposes of this study. The model involves 3‐year moving average values for the base year, 2005. These values were proportionally adjusted for cowpea production, consumption, and trade in west and central Africa, following estimations from Langyintuo and Lowenberg‐DeBoer (2003) who showed that Nigeria is net importer of cowpea. Without the adjustment, Nigeria is self‐sufficient for cowpea in IMPACT. In addition, exogenous yield growth rates, called intrinsic productivity growth rates (IPRs) in IMPACT, were adjusted using data from FAOSTAT to capture the substantial increase in soybean and maize production between 2005 and 2011 in SSA.

In IMPACT, soybean production is classified as both traded and nontraded, and tradability in the model is defined endogenously depending on the differences between international and domestic prices. Nontraded soybean is entirely allocated to domestic processing and is priced based on national supply and demand. Traded soybean is priced based on international markets and can be allocated to various uses, including processing (Robinson et al., 2015).

### 3.1 Climate change models

Two climate change scenarios are used in this study. The first one (NoCC) is optimistic and involves perfect climate change mitigation where the 2050s climate is assumed to be the same as that of the 2000s. The second climate change scenario is more pessimistic and involves the combination of a global Representative Concentration Pathway (RCP) involving a high greenhouse gas emission pathway (RCP 8.5) and the Hadley Global Environment Climate Model (HadGEM2‐ES). Three GCMs (IPSL‐CM5A‐LR, MIROC‐ESM‐CHEM, and HadGEM2‐ES) combined with all RCPs cover the range of simulated future rainfall changes and also have the strongest temperature response to greenhouse gas emissions; among all three, HadGEM2‐ES is the driest (Wiebe et al., 2015). The extreme climate change scenario used in this study involves the drier climate model, HadGEM2‐ES, because soybean is sensitive to water stress. In addition, RCP 8.5 when coupled with HadGEM2‐ES leads to higher global temperatures and more future rainfall anomalies compared to other RCPs involving lower global greenhouse gas emissions (Warszawski et al., 2014).

### 3.2 Socioeconomic scenarios

In this study, projections on the socioeconomic status of national economies by 2050 are based on the Shared Socioeconomic Pathways (SSP; O'Neill et al., 2014). There are five SSPs, reflecting differing assumptions on countries' population, income and other characteristics; differences in these variables across the SSPs are inputs in IMPACT. Two SSPs involve moderate growth in population and income: SSP2 and SSP4. However, SSP4, which is used in this study, most closely matched the degree of income inequality currently observed between African countries.

### 3.3 Promising soybean technologies and adoption pathways

Various innovation scenarios are assessed which vary from improved varieties to policies affecting the processing industry:

- Technology package: combination of improved high‐yielding varieties and improved agronomic practices that double yields and hence are attractive enough to be adopted (at most) on 50% of soybean area (DbleYld)
- Industrial policy: processing policies that lead to a doubling in size of the processing industry and hence of the crushing capacity between 2020 and 2025 (DbleProc)
- Agricultural policy: policies which provide an incentive to farmers for doubling the crop area between 2020 and 2025 (DbleArea)

All innovation scenarios are compared with a base technology scenario (BaseTech) embedded into IMPACT. Under the BaseTech scenario, future investments in research and development are assumed to continue to grow at rates similar to those of the past. Crop yield growth rates are expected to be higher in the developing than the developed world. In addition, these rates are projected to decrease over time to reflect a slowdown in technological improvements (Robinson et al., 2015).

For the innovation scenario DbleYld, we assume adoption pathways which consider the institutional differences across countries relative to seed regulation. In Nigeria, South Africa, and Zambia, the process of releasing improved varieties is much quicker than in other countries. Hence, we assume that the yield‐enhancing technologies would be first released there and much later in the other countries. In addition, the speed of adoption was also assumed to vary across countries based on institutional factors (Table 2).

**Table 2:** Adoption pathways from yield‐enhancing soybean technologies

Country	Time until improved varieties are fully registered, multiplied, and ready for dissemination to farmers (year)	Median year for adoption (half of adoption on soybean acreage has taken place) (year)	Year when maximum adoption occurs (year)
Major producers			
Nigeria	2008	2016	2030
South Africa	2008	2016	2030
Uganda	2009	2020	2030
Zambia	2008	2016	2030
Malawi	2009	2022	2030
Zimbabwe	2009	2022	2030
Rwanda	2010	2026	2030
Ethiopia	2009	2022	2030
Burkina Faso	2010	2026	2030
Benin	2009	2022	2030
Cameroon	2009	2022	2030
Other producers	2010	2026	2030

*Source*: Authors' knowledge.

The technology package that would double yields could consist of improved varieties combined with improved agronomic practices including the use of inoculants, phosphorus application, and the combination of organic and mineral fertilizers. Multiple studies have shown how improved agronomic practices can double soybean yields on farmers' fields in Benin (Zoundji, Houngnandan, Amidou, Kouelo, & Toukourou, 2015), Zimbabwe (Kanonge, Nezomba, Chikomo, Mtambanengwe, & Mapfumo, 2009), and northern Tanzania (Ndakidemi, Dakora, Nkonya, Ringo, & Mansoor, 2006). In addition, maize–soybean rotation or monocrop soybean was proven to more than double soybean yields compared with intercropped soybean and maize in field trials conducted in central Kenya (Herrmann, Chotte, Thuita, & Lesueur, 2014). In IMPACT, the innovation “DbleYld” was modeled by inputting a value of 100% for soybean to the variable “YldTechGR” which reflects growth rates linked to crop technologies.

For the innovation DbleProc, policies would be needed to enhance market infrastructure so as to double the processing capacity across SSA. One policy instrument could consist of reducing the cost of credit access to facilitate investments by the private sector into processing activities. Similarly, doubling acreage could occur through enforcing a floor price for soybean that would incentivize farmers to increase the area planted to the crop. In IMPACT, the innovation was inputted by adjusting “QSINT2,” a growth index for processed products which is used to represent the growth rate in the capacity of the processing industry. For soybean oil and cake, “QSINT2” was adjusted to double between 2020 and 2025 and from 2025, to grow based on its long‐term annual growth rate in the BaseTech scenario.

The innovation DbleArea would be best implemented through market incentives which would make it attractive for farmers to increase soybean acreage. In IMPACT, the innovation is implemented by adjusting the variable “AreaInt2,” an area growth index, and ensuring that it doubles between 2020 and 2025 for soybean.

## 4 RESULTS—PLAUSIBLE FUTURES OF SOYBEAN IN SSA

### 4.1 Soybean futures in SSA without climate change

**Figure 3:**
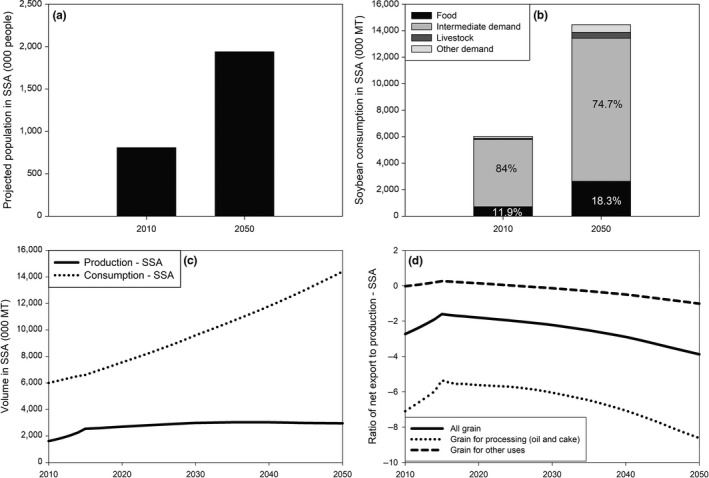
Projected (a) population in SSA by 2050; (b); soybean uses in SSA by 2050; (c) soybean consumption and production in SSA; and (b) soybean import dependency in SSA. *Source*: Authors' computations using results from IMPACT 3.2

Assuming moderate growth in population and income alone, soybean consumption is projected to more than double by 2050 in SSA, from a volume of about 6 million t in 2010 (Figure 3a). More specifically, total consumption would be 2.4 times higher in 2050 than in 2010; this increase would be in line with that of population which is also projected to be 2.4 times higher by 2050 (Figure 3b). Hence, with an annual value of 7.5 g, average consumption per capita would not change in SSA between 2010 and 2050; it would still remain below 36 g, the worldwide average per capita consumption of soybean in 2010.

However, uses would change somewhat between 2010 and 2050. In 2010, 84% of the soybean grain consumed in the region was used as an intermediate product and transformed into oil and cake. By 2050, this share would decrease to 74.7%. However, direct consumption would substantially increase from 11.9% to 18.3% over the same period. This increase in direct consumption would suggest a diet change where soybean would play a more prominent role as a protein source by 2050 (Figure 3b).

Soybean production in SSA would not keep up with consumption between 2010 and 2050. Production is projected to increase by 83% between 2010 and 2050; it would even reach a peak of 3.04 million t around 2040 before decreasing slightly until 2050. Hence, the demand gap which stood at 4.4 million t in 2010 would more than double to 11.4 million by 2050 (Figure 3c).

The increasing dependence on imports is better illustrated through the ratio of net export to domestic production. For net importers, this ratio is negative; hence, an increase in this ratio translates into import substitution where domestic production replaces imports. In 2010, soybean net imports in SSA were 2.7 times higher than its domestic production. However, the region was mainly dependent on imports for processed soybean (oil and cake). Net imports of oil and cake were 7 times higher than their domestic production in 2010. The region was self‐sufficient in terms of the soybean grain involved in other uses apart from processed oil and cake: the net imports of grain for other uses were almost nil when compared to domestic grain production. However, by 2050, soybean import dependency is projected to worsen in the region. The net imports of processed products (oil and cake) would be 8.6 times higher than their domestic production. The ratio of net imports to domestic production for other uses would reach −1; this would imply that the region would find itself importing a volume of soybean for other uses that would be equivalent to its domestic production by 2050 (Figure 3d).

### 4.2 Impact of climate change on soybean futures for SSA

**Figure 4:**
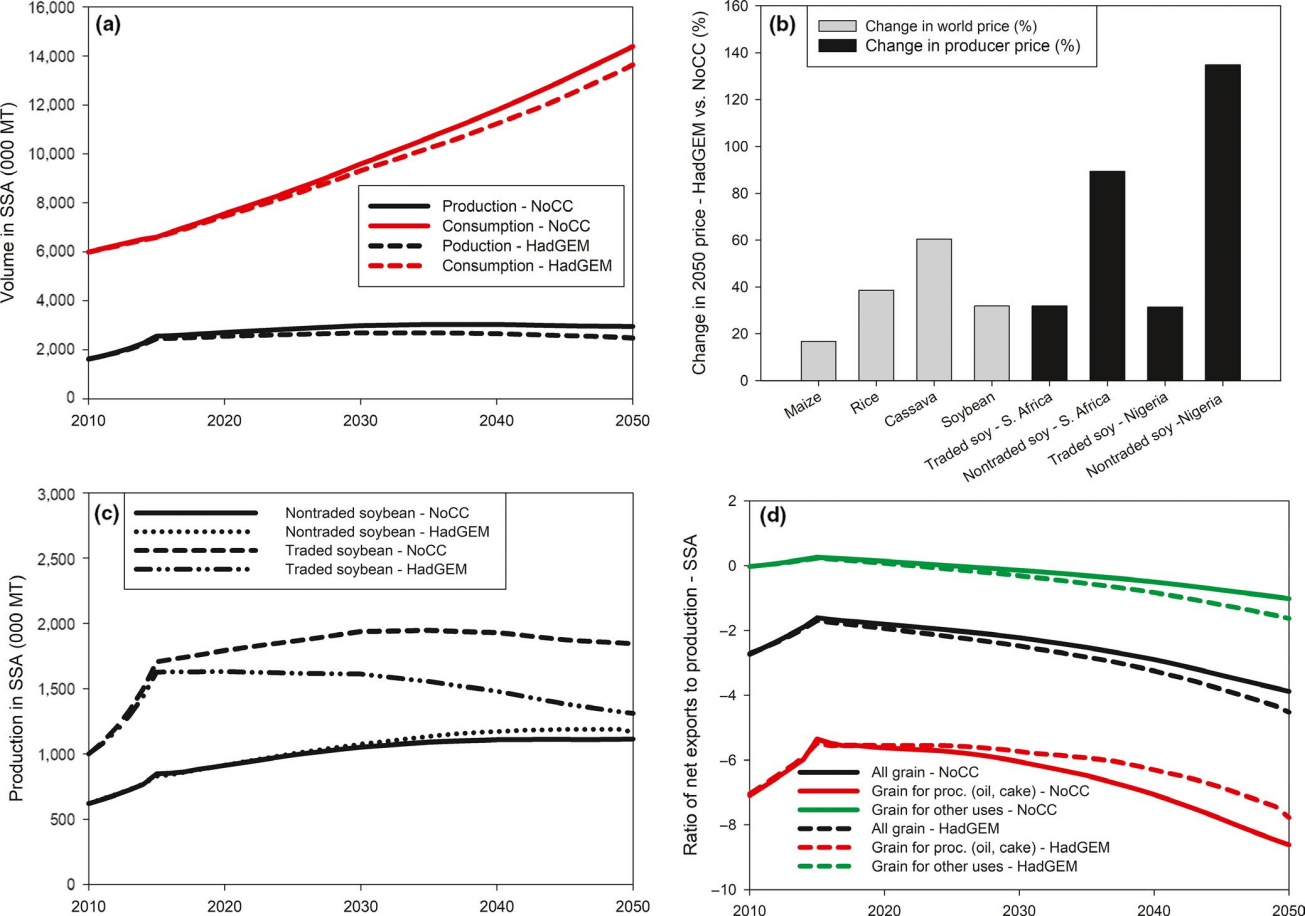
Projected (a) trends in soybean production and consumption in SSA under climate change; (b) prices for key staple crops in SSA; (c) trends in the production of traded and nontraded soybean in SSA; and (d) soybean import dependency under climate change in SSA. *Source*: Authors' computations using results from IMPACT 3.2

Under the drier climate change model, HadGEM2‐ES, soybean production in SSA would decrease by 16% in 2050 compared with the base climate scenario, NoCC (Figure 4a). Such reduction reflects the bio‐economic impact of climate change, a combination of the biophysical and socioeconomic effects on soybean yields. Climate change would have a physiological impact where higher temperatures and reduced rainfall would decrease yields. However, climate change will also affect the yields of other food crops, including maize, rice, and cassava which are key staples in SSA; this will affect crop prices and in turn indirectly influence soybean production and consumption (Islam et al., 2016).

For example, by 2050 under the climate change scenario using the HadGEM climate model and RCP 8.5, world prices would increase for maize by 17%, rice by 39%, and cassava by 60%, compared with the base scenario, NoCC (Figure 4b). These changes would be expected to influence farmers' allocation of agricultural inputs, including land, toward soybean production. The increased scarcity would also increase the price of non‐(internationally)‐traded soybean, and the latter should experience a higher price increase than traded soybean. This is reflected in South Africa and Nigeria, the two largest soybean producers in SSA. In both countries, the producer price for traded soybean would be 32% higher in 2050 under the climate change scenario compared with the base scenario, NoCC. However, for nontraded soybean, the producer price increase would be 89% in South Africa and 135% in Nigeria.

For SSA, the effect of climate change on the prices of nontraded soybean would be substantial enough to increase production despite the negative biophysical effect of climate change on yields. Hence, the production of nontraded soybean is projected to increase by 5% in 2050 under the climate change scenario compared with the base scenario, NoCC. Such increase would be associated with a decrease of 30% in the production of traded soybean in 2050 (Figure 4c).

Climate change would also reduce soybean consumption in SSA (Figure 4a). By 2050, the consumption of processed products (oil and cake) would decrease by 5% under the climate change scenario compared with the base scenario, NoCC. For other uses, the decrease would be 7%. In volume terms, the reduction in total consumption (778 thousand t in 2050) would be larger than that of production (481 thousand t in 2050). Hence, net soybean imports would decrease by about 296 thousand t because of climate change. However, the reduction in net imports would be smaller than the reduction in production, leading to an increase in soybean import dependency, as reflected by a decrease in the ratio of net export to domestic production for all soybean grain under climate change (Figure 4d).

For the grain used in processing oil and cake, the import dependency would slightly reduce under climate change. The increased production of nontraded soybean in SSA combined with a reduction in the consumption of oil and cake would lead to some import substitution. Hence, by 2050, the ratio of net exports to domestic production for processed soybean would reach −7.8 under the HadGEM2‐ES climate change model (Figure 4d).

### 4.3 Impact of soybean innovations on soybean futures in SSA

**Figure 5:**
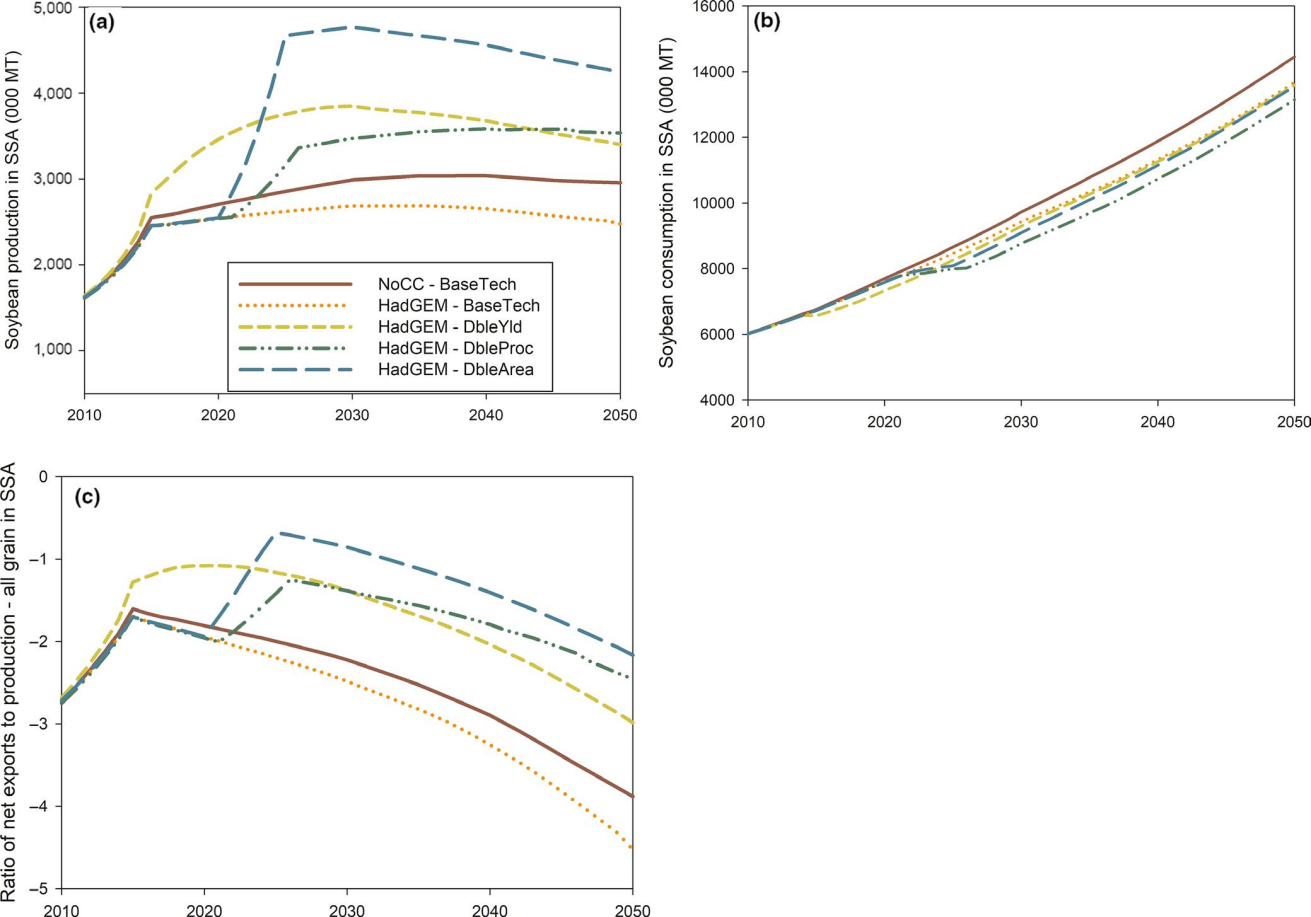
Projected impact of soybean innovations on (a) soybean production in SSA; (b) soybean consumption in SSA; and (c) terms of trade for soybean in SSA. *Source*: Authors' computations using results from IMPACT 3.2

The soybean innovations would substantially increase production (Figure 5a) and, by 2050, would lead to an increase of 37% (HadGEM‐DbleYld), 43% (HadGEM‐DbleProc), and 71% (HadGEM‐DbleArea) in soybean production compared with the scenario involving base technologies under climate change (HadGEM‐BaseTech). In addition, under each of these alternative innovations, production by 2050 would exceed that projected under the scenario involving no climate change (NoCC‐BaseTech; Figure 5a).

On the other hand, the alternative innovations would not lead to consumption exceeding that projected in the scenario involving no climate change (NoCC‐BaseTech). For two innovations, (DbleYld and DbleArea), consumption in 2050 under climate change would be similar to that projected under the scenario involving base agricultural technologies (HadGEM‐BaseTech). For the scenario involving a doubled size of the processing industry (HadGEM‐DbleProc), soybean consumption in 2050 would be slightly smaller than that projected under the base technologies (HadGEM‐BaseTech; Figure 5b).

The increased production brought by the alternative innovations coupled with consumption which would either remain the same or decrease implies a reduction in import dependency (Figure 5c). However, even under the innovation which provides the highest production increase, (DbleArea), net imports into SSA would still be more than twice the domestic production by 2050.

Each innovation would influence production through specific mechanisms which need to be understood to identify the most appropriate combination of innovations needed to eliminate import dependency in SSA. The innovation DbleArea would directly affect acreage in SSA but would have indirect effects on the production, prices, and yields of soybean and other food crops. An increase in soybean acreage, ceteris paribus, should increase production through a supply shift and hence lower producer and consumer prices. For nontraded soybean, the reduction in prices should be higher than that for traded soybean, given that most countries in Africa are world price‐takers for traded soybean. The reduction in consumer prices should increase the consumption of soybean, and this should be accompanied by some decrease/increase in the consumption of other food products which are substitutes/complements to soybean‐based food products. In response to the changing demands, agricultural inputs, including fertilizer and labor, would be reallocated across crops, including soybean, and this would shift production levels. The shifts in production and demand for various crops would stabilize over time to generate long‐term changes in production, consumption, and trade which are reflected in IMPACT.

The innovation would also affect crop yields in SSA through changes in the allocation of irrigation water. In IMPACT, crops' economic value is a criterion used in the allocation of irrigation water. Given that soybean is produced with supplemental irrigation in some parts of Africa, the innovation DbleArea would increase the demand for irrigation water allocated to soybean. In some instances, not enough water would be supplied and this could lead to a decrease in yields for soybean and/or other crops. In addition, the innovation would influence crop yields through changes in input use as African producers would have to allocate a greater portion of their scarce inputs, including fertilizer and labor, toward soybean.

All in all, the innovation DbleArea would have different effects across traded and nontraded soybean. For traded soybeans, acreage in SSA would slightly more than double by 2050 compared to the scenario involving base agricultural technologies and policies (HadGEM‐BaseTech; Table 3). Moreover, the change in acreage would be accompanied by a similar change in production. However, variations would exist across countries. For example, in Côte d'Ivoire and Zambia, the policy would lead to both soybean acreage and production tripling by 2050. Traded soybean acreage without the innovation (HadGEM‐BaseTech) would stand in 2050 at around 0.04% of total crop acreage for Côte d'Ivoire and 0.13% for Zambia. Hence, increasing soybean acreage in these two countries would not come at a huge cost in the acreage and production of other crops. Even for Nigeria, the largest producer of traded soybean in SSA by 2050 under the HadGEM‐BaseTech scenario, soybean acreage in 2050 without the innovation would stand at about 0.77% of total crop acreage. Under the innovation, crops that would experience the largest reductions in acreage in Nigeria are maize with −1%; other pulses with −1.2%; and other traded oilseeds with −1.2%. Only two crops would experience an increase in their acreage under the innovation: yam with 0.1% and nontraded groundnut with 0.3% (Table 3).

**Table 3:** Impact of soybean innovation "DbleArea" on yield, acreage, production, and producer prices in SSA under a drier future climate

	Change under HadGEM‐DbleArea vs. HadGEM‐BaseTech in 2050 (%)							
	Traded soybean				Nontraded soybean			
	Yield	Acreage	Production	Producer price	Yield	Acreage	Production	Producer price
Burundi	0	109	109	−1	–	–	–	–
Benin	0	171	170	−1	−8	89	73	−970
Burkina Faso	0	176	175	−1	–	–	–	–
Côte d'Ivoire	0	196	196	−1	–	–	–	–
Cameroon	0	162	162	−1	–	–	–	–
Congo, DR	0	118	118	−1	–	–	–	–
Ethiopia	−3	83	78	−1	–	–	–	–
Gabon	–	–	–	–	−12	84	62	−83
Kenya	0	128	127	−1	–	–	–	–
Liberia	0	108	108	−1	–	–	–	–
Madagascar	0	139	138	−1	–	–	–	–
Mali	0	128	128	−1	–	–	–	–
Malawi	−1	125	123	−1	–	–	–	–
Nigeria	0	117	116	−1	−3	47	42	−39
Rwanda	0	108	108	−1	–	–	–	–
Tanzania	0	175	175	−1	–	–	–	–
Uganda	0	115	115	−1	−11	31	16	−72
South Africa	−5	127	115	−1	−11	19	6	−70
Zambia	0	224	223	−1	0	181	181	−151
Zimbabwe	–	–	–	–	−11	125	99	−72
SSA	−1	119	116		−17	45	21	

Note

indicates not applicable for country.

*Source*: Authors' computations using results from IMPACT 3.2.

For nontraded soybean, the innovation DbleArea would increase soybean acreage and production in SSA by 45% and 21%, respectively, compared with the HadGEM‐BaseTech scenario. Here, the reductions in producer prices brought by the innovation would be substantial enough to prevent producers from fully doubling acreage. In Uganda, the second largest producer of nontraded soybean in SSA by 2050 under the HadGEM‐BaseTech scenario, the innovation would lead to a long‐term decrease of 72% in the producer price. Such reduction would be linked to an increase of 31% in nontraded soybean acreage. The innovation would also reduce nontraded soybean yields by 11%, and this would be caused solely by a reduction in the amount of agricultural inputs, including fertilizer and labor, allocated per soybean acre. Hence, nontraded soybean production in Uganda would increase by 16% only with the innovation (Table 3).

In Zambia, despite a reduction of 151% in the producer price, nontraded soybean acreage and production would nearly triple (Table 3). This would occur in part because nontraded soybean is not an important crop in the country. The share of nontraded soybean in total crop acreage would be 0.12% by 2050 under the HadGEM‐BaseTech scenario. Hence, increasing soybean acreage would not come at a high cost for producers in Zambia. A similar situation is seen with Zimbabwe where nontraded soybean acreage would more than double, with production doubling despite a 72% reduction in producer price (Table 3). In Zimbabwe, the share of nontraded soybean without the innovation (HadGEM‐BaseTech) would reach 0.83% of total crop acreage by 2050.

The innovation which will lead to the second highest increase in production is DbleProc. Doubling the size of the processing industry, ceteris paribus, should increase the domestic demand for soybean grain used in processing. Hence, producer prices should increase and this should increase domestic production. The larger size of the processing industry should also translate into an increased production of oil and meal. This increased supply should influence the demand of other food products; in response, producers and processors would readjust food production levels. Hence, the innovation would also have indirect effects on the production and consumption of soybean and other food products.

In the long term, the intermediate demand for soybean would nearly double in 2050 under the HadGEM‐DbleProc scenario compared with the HadGEM‐BaseTech scenario for SSA. In some countries, Benin and Zambia, the demand for soybean grain used for processing would more than double under the HadGEM‐DbleProc scenario compared with HadGEM‐BaseTech scenario in 2050. However, for the countries with the highest demand for soybean grain used in processing, namely South Africa and Uganda, the increase would be 96% and 66%, respectively. The increase in the volume of grain used in processing would lead to corresponding increases in the production of oil and meal across SSA (Table 4).

**Table 4:** Impact of soybean innovation "DbleProc" on yield, acreage, production, and producer prices in SSA under a drier future climate

	Change under HadGEM−2Proc vs. HadGEM‐BaseTech in 2050 (%)									
	Soybean volume—processing	Soybean oil/meal production	Traded soybean				Nontraded soybean			
			Soybean yield	Soybean acreage	Soybean production	Producer price	Soybean yield	Soybean acreage	Soybean production	Producer price
Burundi	–	–	0	0	0	0	–	–	–	–
Benin	153	153	0	0	0	0	21	108	153	1515
Burkina Faso	–	–	0	0	0	0	–	–	–	–
Côte d'Ivoire	–	–	0	0	0	0	–	–	–	–
Cameroon	–	–	0	0	0	0	–	–	–	–
Congo, DR	–	–	0	0	0	0	–	–	–	–
Ethiopia	–	–	0	0	1	0	–	–	–	–
Gabon	46	46	–	–	–	–	6	26	34	141
Kenya	–	–	0	0	0	0	–	–	–	–
Liberia	–	–	0	0	0	0	–	–	–	–
Madagascar	–	–	0	0	0	0	–	–	–	–
Mali	–	–	0	0	0	0	–	–	–	–
Malawi	–	–	0	0	0	0	–	–	–	–
Nigeria	49	49	0	1	1	0	3	45	49	60
Rwanda	–	–	0	0	0	0	–	–	–	–
Tanzania	–	–	0	0	0	0	–	–	–	–
Uganda	66	66	0	0	0	0	9	52	66	143
South Africa	96	96	0	0	0	0	10	79	96	205
Zambia	272	272	0	0	0	0	42	162	272	230
Zimbabwe	39	39	–	–	–	–	5	18	24	75
SSA	90	92	0	1	1		21	57	90	

Note

indicates not applicable for country.

*Source*: Authors' computations using results from IMPACT 3.2.

All nontraded soybean produced in SSA is allocated to processing; in addition, Gabon and Zimbabwe import a small amount to use in the processing industry. The innovation would increase producer prices for nontraded soybean across SSA. The smallest increase would occur in Nigeria with 60%. The highest would occur in Benin where producer prices would increase by more than 10 times under the HadgEM‐DbleProc scenario compared with the HadGEM‐BaseTech scenario in 2050. The increase in producer prices would be linked to a higher production of nontraded soybean under the HadGEM‐DbleProc scenario compared with the HadGEM‐BaseTech scenario. Moreover, the production increase would be similar to that of the volume of soybean grain allocated to the processing industry: both would increase by 90% across SSA under the HadGEM‐DbleProc scenario compared with the HadGEM‐BaseTech scenario. The innovation would not affect producer prices for traded soybean and hence would have no impact on its production (Table 4).

With the last innovation, DbleYld, if the improved technology which doubles yield under the HadGEM‐DbleYld is adopted on 50% of soybean acreage across SSA, total production in the region should increase by 50% compared with the HadGEM‐BaseTech scenario. However, the increase is only 37% by 2050 (Table 5). The key reason for the discrepancy relates to the production of nontraded soybean.

**Table 5:** Impact of soybean innovation "DbleYld" on soybean yield, acreage, production, and producer prices in SSA under a drier future climate

	Change under HadGEM‐DbleYld vs. HadGEM‐BaseTech in 2050 (%)							
	Traded soybean				Nontraded soybean			
	Yield	Acreage	Production	Producer price	Yield	Acreage	Production	Producer price
Burundi	50	7	60	−1	–	–	–	–
Benin	40	6	48	−1	30	−18	7	−66
Burkina Faso	41	6	49	−1	–	–	–	–
Côte d'Ivoire	47	6	56	−1	–	–	–	–
Cameroon	45	6	54	−1	–	–	–	–
Congo, DR	50	7	60	−1	–	–	–	–
Ethiopia	41	9	54	−1	–	–	–	–
Gabon	–	–	–	–	40	−7	31	−46
Kenya	48	6	58	−1	–	–	–	–
Liberia	50	7	62	−1	–	–	–	–
Madagascar	51	7	61	−1	–	–	–	–
Mali	50	7	60	−1	–	–	–	–
Malawi	46	6	55	−1	–	–	–	–
Nigeria	49	20	79	−1	45	−5	38	−36
Rwanda	45	6	54	−1	–	–	–	–
Tanzania	48	6	57	−1	–	–	–	–
Uganda	45	6	54	−1	39	−18	15	−72
South Africa	43	12	60	−1	34	−23	4	−57
Zambia	37	5	44	−1	37	8	48	−68
Zimbabwe	–	–	–	–	44	−2	41	−36
SSA	40	15	61		31	−16	10	

Note

indicates not applicable for country.

*Source*: Authors' computations using results from IMPACT 3.2.

The improved technology would increase production, ceteris paribus, since it would allow farmers to grow more on the same amount of land. The increased production would be accompanied by a reduction in market prices and consequently producer prices. The increased production and consumption should be accompanied by consumption changes for other goods which are substitutes or complements to soybean‐based food products. Changes in the consumption of other products would affect their production and hence the allocation of resources toward agricultural production. In the long term, consumer and producer prices for traded soybean should not decrease as much as those for nontraded soybean. However, the change in production should be more pronounced for traded than for nontraded soybean.

In the long run, producer prices would decrease by 1% for traded soybean; however, for nontraded soybean, the decrease would be more pronounced, ranging between 35% and 75% (Table 5). For nontraded soybean, the acreage would decrease but the yield increase brought by the improved technology would overcompensate for that decrease. The decrease in the acreage of nontraded soybean would be reflecting the fact that farmers can grow more on the same amount of land. However, the increased yield for nontraded soybean would be smaller than 50%, partly because farmers would reduce the allocation of other inputs, such as fertilizer and labor, to nontraded soybean. Hence, with this innovation, nontraded soybean yields would increase by 30%–45% across SSA (Table 5).

For traded soybean, the reduction in producer prices would be minimal. Hence, farmers would have stronger incentives to increase their traded soybean production. The acreage for traded soybean would increase with the innovation. Similarly, yields would increase, although in most countries they would increase by <50%. Here, too, farmers would reduce their allocation of some inputs such as fertilizer and labor to soybean production once they adopt the improved technology. The increase in both yield and acreage would lead to production increasing by more than 60% in SSA. Nigeria, the second largest soybean producer in 2050 under the HadGEM‐BaseTech scenario, would experience the largest production increase (79%) with the innovation (Table 5).

### 4.4 Soybean innovations to reduce import dependency

Based on the above analysis, three additional combinations of innovations are assessed: DbleYldArea which combines DbleYld and DbleArea; DbleYldAreaProc which combines DbleYld, DbleArea, and DbleProc; and DbleAreaProcYldAdopt75 which combines doubling soybean area (DbleArea), doubling the size of the processing industry (DbleProc) and introducing a technology package which would double yields and be adopted on 75% of soybean acreage in SSA.

**Figure 6:**
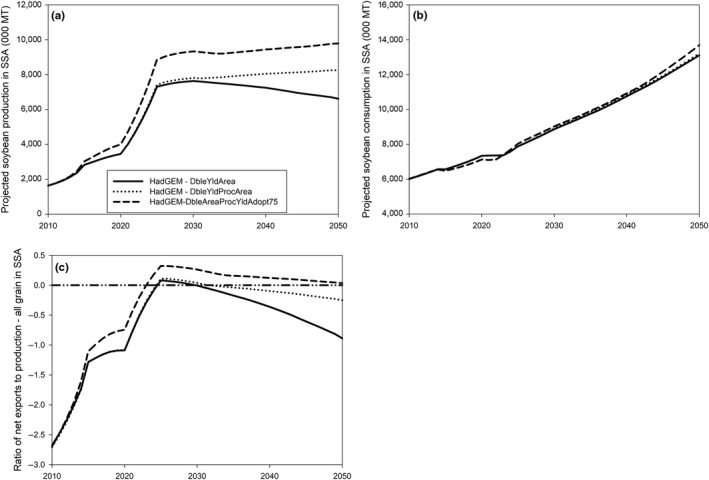
Projected impact of soybean innovation mix on (a) soybean production in SSA; (b) soybean consumption in SSA; and (c) terms of trade for soybean in SSA. *Source*: Authors' computations using results from IMPACT 3.2

All three combinations of innovations would have substantial positive effects on production. More specifically, by 2050, production in SSA would be 2.7 times higher under the DbleYldArea scenario compared with the HadGEM‐BaseTech scenario; it would be 3.3 times higher under the DbleYldAreaProc scenario and would nearly quadruple under the DbleAreaProcYldAdopt75 scenario. On the other hand, the technologies would not affect consumption in the region. Among all three combinations of innovations, the last one, DbleAreaProcYldAdopt75, would allow SSA to become self‐sufficient for soybean by 2023 and remain self‐sufficient up to 2050 (Figure 6).

## 5 DISCUSSION

Substantial progress has been achieved in terms of developing soybean germplasm that delivers higher yields for farmers in SSA. The annual genetic gain from improved germplasm developed over 16 years of breeding research in Africa varies from 2.2% for early maturing varieties to 1.42% for late maturing varieties (Tefera, Asafo‐Adjei, & Dashiell, 2010; Tefera, Kamara, Asafo‐Adjei, & Dashiell, 2009). Despite these achievements, domestic production in SSA has not kept up with demand over the years. The geospatial bio‐economic framework used in this study implies that the demand gap for soybean in SSA is projected to substantially increase over time under two future climates, one involving perfect climate change mitigation and the other a drier climate. Our results are consistent with earlier studies on the impact of climate change on soybean production. Adhikari, Nejadhashemi, and Woznicki (2015) estimated that the increased temperature brought by long‐term climate change could lead up to a 10% reduction in soybean yields in eastern and southern Africa by 2050. However, climate change could also severely affect soybean yields in SSA through emerging biotic stresses (Pimentel, 1993). Hence, this study provides conservative estimates on the holistic impact of climate change on yields.

This study also shows that a combination of bold innovations would be needed to close the demand gap in SSA by 2050 under a drier future climate. These innovations would need to deliver yield gains which are much higher than those obtained through conventional breeding. One such mix of innovations would involve doubling soybean acreage and the size of the processing industry by 2025; it would also involve ensuring that a technology package which doubles yields is adopted on 75% of soybean acreage across SSA. A range of improved cultivars and agronomic practices have been identified which can double yields on farmers' fields across SSA (Herrmann et al., 2014; Kanonge et al., 2009; Ndakidemi et al., 2006; Zoundji et al., 2015). However, proper targeting of these technologies would be required to ensure that adequate technology packages are defined for each recommendation domain in SSA. Process‐based biophysical models could facilitate such an endeavor. In addition, the yield‐enhancing technology packages would need to be adopted across SSA and this would call for better functioning seed systems and national agricultural extension systems.

Strengthening the processing sector across SSA would require addressing locally specific constraints. For example, in Nigeria, restrictive trade policies are currently in place with an aim of protecting the burgeoning processing industry. However, processors in Nigeria face other constraints which limit investments: high energy costs, limited access to credit, and difficult access to good quality grain for processing (Nzeka, 2014). In southern Africa, constraints faced by the oilseed processing industry include high transport costs, informal trade barriers which delay product delivery, and a high ratio between processing capacity and soybean grain availability which results in a low level of utilization of oilseed processing equipment (Opperman & Varia, 2011).

Doubling soybean acreage across SSA might also require policy changes in some countries. Current acreage in SSA is low, and this study demonstrates how doubling this acreage between 2020 and 2025 would not come at a substantial reduction in crop acreage for other crops. One way to incentivize farmers to increase soybean acreage would be for policy‐makers to strengthen the oilseed processing industry and ensure that it offers attractive producer prices. Another avenue would be contract farming, with an enforced floor price for soybean, which would allow resource‐poor farmers to produce quality grain for processing while receiving adequate support for production. Similarly, the occurrence of dry spells, especially in southern Africa (Fauchereau, Trzaska, Rouault, & Richard, 2003), would call for investment in irrigation infrastructure to support soybean production.

Soybean in Africa is a cash crop; as such, enhancing domestic soybean production should influence farmers' income and hence their production and investment decisions in subsequent years. Similarly, the income changes brought by the innovations should influence poverty and food security outcomes. Unfortunately, the economic model used in this study involves partial equilibrium and hence does not consider income feedback effects. A global CGE model would be needed to quantify the net effect of the innovations on income, poverty, and production decisions. However, the direction of the changes brought by the innovations should remain the same with a CGE model.

In addition, soybean raises soil fertility and boosts yields for subsequent crops; it has been shown that the adoption of the soybean–maize rotation in northern Nigeria led to maize yields doubling over time (Akinola, Alene, Adeyemo, Sanogo, & Olanrewaju, 2009). Incorporating such positive effects in IMPACT would have shown the double role of the innovations: reduce demand gap and enhance food and nutrition security in the region through the positive impact of the yields of other crops.

## 6 CONCLUSION

This study uses a global geospatial bio‐economic model to analyze plausible soybean futures for SSA. Projections imply a widening of the current soybean demand gap by 2050 under two climate change models, an optimistic one involving perfect climate change mitigation till 2050 and a pessimistic one involving a drier future climate (HadGEM2‐ES). More specifically, net soybean imports into SSA would be more than four times higher than domestic production by 2050 under a future drier climate. Bold innovations calling for a combination of policies affecting the soybean value chain would be needed to reduce the demand gap by 2050. They involve an increase of at least 100% in the size of the processing industry and in both yields and acreage on farmers' fields. Only the abiotic stresses brought by climate change are considered in this study, and yet emerging pests and diseases brought by climate change could severely affect future yields in SSA. Hence, further work should incorporate biotic stress modeling and attempt to quantify the holistic impact of climate change on yields across SSA. In addition, a global CGE model should be used to quantify the net effect of these innovations on poverty.
